# Brown locusts, *Locustana pardalina*, host fluconazole-resistant *Candidozyma* (*Candida*) *auris*, closely related to Clade III clinical strains

**DOI:** 10.1093/mmy/myaf069

**Published:** 2025-07-24

**Authors:** Adepemi Ogundeji, Maryam Bello-Akinosho, Vaughn Swart, Jonathan Featherston, Errol D Cason, Armand Bolsenbroek, Carel Beneke, Jolly Musoke, Tyla Baker, Arshad Ismail, Olihile Sebolai, Jacobus Albertyn, Carolina Pohl

**Affiliations:** Department of Microbiology and Biochemistry, University of the Free State, Bloemfontein, 9301, South Africa; Department of Microbiology and Biochemistry, University of the Free State, Bloemfontein, 9301, South Africa; Department of Zoology and Entomology, University of the Free State, Bloemfontein, 9301, South Africa; Sequencing Core Facility, National Institute for Communicable Diseases a Division of the National Health Laboratory Service, Sandringham, 2192, South Africa; Department of Animal Sciences, University of the Free State, Bloemfontein, 9301, South Africa; Department of Microbiology and Biochemistry, University of the Free State, Bloemfontein, 9301, South Africa; Department of Microbiology and Biochemistry, University of the Free State, Bloemfontein, 9301, South Africa; Department of Medical Microbiology, School of Pathology, Faculty of Health Sciences, University of the Free State, Bloemfontein, 9301, South Africa; National Health Laboratory Service, Department of Medical Microbiology, Universitas Academic Hospital, Bloemfontein, 9301, South Africa; Department of Microbiology and Biochemistry, University of the Free State, Bloemfontein, 9301, South Africa; Sequencing Core Facility, National Institute for Communicable Diseases a Division of the National Health Laboratory Service, Sandringham, 2192, South Africa; Department of Biochemistry and Microbiology, Faculty of Science, Engineering and Agriculture, University of Venda, Thohoyandou, 0950, South Africa; Department of Microbiology and Biochemistry, University of the Free State, Bloemfontein, 9301, South Africa; Department of Microbiology and Biochemistry, University of the Free State, Bloemfontein, 9301, South Africa; Department of Microbiology and Biochemistry, University of the Free State, Bloemfontein, 9301, South Africa

**Keywords:** *Candidozyma* (*Candida*) *auris*, *Locustana pardalina*, environmental niche, fluconazole, disinfectants, stress tolerance, One Health

## Abstract

The environmental niche and mode of transmission from the environment to humans of the emerging pathogenic yeast, *Candidozyma* (*Candida*) *auris* is a subject of speculation, with hypotheses including avian species and marine environments. Interestingly, yeasts related to *Candidozyma auris* have been repeatedly observed associated with various insects. This prompted us to investigate a thermophilic insect, *Locustana pardalina*, as a possible host for *C. auris*. Here, we report the isolation and identification of three *C. auris* strains from the gut of *L. pardalina* as well as the phenotypic characterisation of one of these isolates. Interestingly, the isolate was able to survive at 50°C and grew at 15% NaCl. In addition, it was susceptible to the tested disinfectants and antifungals, except fluconazole. Genome sequencing and single-nucleotide polymorphism analyses placed the isolate in Clade III, which is common in South African hospitals. This highlights the potential role of thermotolerant insects in the evolution and dissemination of emerging pathogenic yeasts.

## Introduction

*Candida auris*, an emerging pathogenic yeast that is able to cause nosocomial outbreaks, forms part of a clade of closely related species, consisting of *Candida auris, Candida duobushaemuli* (also known as *C. duobushaemulonii*)*, Candida haemuli* (also known as *C. haemulonii*)*, Candida haemuli* var. *vulneris, Candida heveicola, Candida khanbhai, Candida konsanensis, Candida metrosideri, Candida ohialehuae, Candida pseudohaemuli* (also known as *C. pseudohaemulonii*)*, Candida ruelliae*, and *Candida vulturna* in the *Metschnikowiaceae*. This classification has recently been revised, and these species have been reassigned to the new genus *Candidozyma*. The updated nomenclature will be used here. Many of these species can cause human infections and are resistant to antifungal drugs.^1^

One of the unanswered questions regarding the emergence of *Candidozyma auris* is its environmental niche and mode of transmission from the environment to humans. It has been hypothesised that this yeast might have intermediate hosts, such as birds, which could facilitate its spread and transmission to humans.^2^ This is speculated to be due to the high body temperature of birds that would allow colonisation or infection only by thermophilic yeasts able to grow at > 40°C, allowing their spread via migratory birds to different geographic locations. However, no evidence is available regarding direct isolation of *C. auris* or the presence of its DNA from birds.^3,4^

Interestingly, members of *Candidozyma* are associated with insects, which may act as vectors of these yeasts. Strains of *C. duobushaemuli* have been isolated from the European firebug (*Pyrrhocoris apterus*)^5^, and its DNA has been detected in microbiomes from the Asian tiger mosquito (*Aedes albopictus*). Similarly, DNA sequences of *C. haemuli* were present in Svensson's copper underwing (*Amphipyra berbera*), the cosmopolitan springtail (*Entomobrya nivalis)*, double-spined bark beetle (*Ips duplicatus*), six-toothed bark beetle (*Ips sexdentatus*), slender springtail (*Orchesella flavescens*), and longhorn crazy ant (*Paratrechina longicornis*). In addition, sequences of *C. haemuli, C. haemuli* var. *vulneris*, and *C. duobushaemuli* were abundant in microbiomes of termites (*Isoptera* spp.), while DNA sequences of *C. heveicola* were detected in samples from the gut of the Julia butterfly (*Dryas iulia*), the ghost yellow butterfly (*Eurema albula*), and the spot-banded daggerwing (*Marpesia merops*).^4^ This insect association prompted us to investigate the presence of *C. auris* (a known thermotolerant yeast) in insects that can withstand high temperatures.

An example of a group of cosmopolitan insects with a high body temperature preference is locusts. Migratory locusts (*Locusta migratoria*) have a preferred body temperature of 38–39°C if food is abundant^6^, although lower temperatures are selected when food becomes limiting.^7^ The desert locust (*Schistocerca gregaria*) can tolerate 50°C without apparent adverse effects.^8^ Similarly, the brown locust (*Locustana pardalina*) is typically exposed to high ambient temperatures of 33–38°C, soil temperatures of 39–62°C, and is reported to have a preferred body temperature of 39–41°C.^9^ Thus, we investigated the presence of *C. auris* in adult *L. pardalina* in South Africa.

## Materials and methods

### Collection of *Locustana pardalina*

Twenty gregarious (swarming) adult locusts were collected by sweep net on 16 April, 2022, during a large locust outbreak, which occurred from September 2021 to May 2022. The coordinates of the sample site are 31°58'45.99‘S and 24°42'7.58’E, within the semi-arid Eastern Karoo climactic region in the Eastern Cape of South Africa. This is a remote rural area, consisting mostly of large private grazing farms with minimal human occupation.

### Yeast isolation from the alimentary canal of brown locust

The locusts were surface sterilised, and the entire alimentary canal was removed and dissected into the fore-, mid-, and hindgut. Each section was vigorously rinsed in sterile distilled water. This rinse water was plated onto Yeast Malt Extract (YM) agar (3 g/l malt extract, 3 g/l yeast extract, 5 g/l peptone powder, 10 g/l d-glucose, and 16 g/l agar) and incubated at 30°C. Colonies with different characteristics were sub-cultured until pure cultures were obtained.

### Identification of isolated strains

All the pure colonies were cultured in YM broth at 37°C overnight while shaking. Two ml of the culture was concentrated using centrifugation (Centrifuge 5430R Eppendorf® USA: F—35–6—30 rotor) and was resuspended in 200 µl of Phosphate Saline Buffer. The complete 200 µl of resuspended cells was used to extract genomic DNA (gDNA) using the protocol stipulated in the ZYMO Research Quick DNA TM Fungal/Bacterial Miniprep Kit (ZymoResearch, USA). gDNA extraction was confirmed using gel electrophoresis (0.8% agarose gel at 90 V, 400 mA for 25 min).

Both the ITS region and the D1/D2 domains of the LSU rRNA gene were amplified by polymerase chain reaction (PCR) using the respective primer pairs of ITS4 (5′-TCCTCCGCTTATTGATATGC-3′) and ITS5 (5’-GGAAGTAAAAGTCGTAACAAGG-3’) as well as NL1 (5′-GCATATCAATAAGCGGAGGAAAAG-3′) and NL4 (5′-GGTCCGTGTTTCAAGACGG-3′), according to Kurtzman and Robnett.^10^ Following amplicon clean-up and post-clean-up PCR, amplicons were sequenced using the BigDye^TM^ Terminator Sequencing kit on the Applied Biosystems 3500 genetic analyser. Consensus sequences obtained for each isolate were compared to the GenBank nucleotide data library using the Basic Local Alignment Search Tool (BLAST) software^11^ at the National Centre for Biotechnology Information (NCBI) website (http://www.ncbi.nlm.nih.gov).

All strains were deposited in the Biodiversity Biobanks South Africa Yeast culture collection, held at the Department of Microbiology and Biochemistry, University of the Free State, and preserved in glycerol stocks at -80°C. Only one of the identified *C. auris* strains (UOFS Y-4024) could be revived from cryostorage and was further characterised.

### Genomic characterisation

Extracted gDNA was quantified using the Qubit 4 Fluorometer with the Qubit™ dsDNA HS Assay Kit (Thermo Fisher Scientific, Waltham, MA, USA). Paired-end library (2 × 150 bp) was prepared using Illumina DNA Prep kit (Illumina, San Diego, US), followed by sequencing on an Element Biosciences Aviti™ Sequencer using Cloudbreak Freestyle chemistry kit (Element Biosciences, San Diego, CA, USA) with 100X coverage.

Raw sequencing reads of *C. auris* UOFS Y-4024 were processed using the v1.5 mycoSNP^12^ Nextflow (v23.10.0) nf-core^13^ pipeline. The Centre for Disease Control *C. auris* reference genome (GCA_016772135.1_ASM1677213v1_genomic.fna) was used for alignment and single-nucleotide polymorphism (SNP) identification. In addition to the study isolates, sequence reads were obtained from the NCBI Sequence Read Archive (SRA) for a total of 32 publicly available *C. auris* genomes from Clades I to VI based on clade-level classification by Suphavilai and co-workers^14^ and included for phylogenetic analyses (Supplementary Table S1). Briefly, (i) the mycoSNP pipeline prepared the reference genome for alignment with nucmer, samtools, Picard tools, and BWA index and (ii) sample QC was assessed with FastQC^15^, poor quality sequence data were trimmed with FaQCS v 2.10 and SeqKit, trimmed sequence reads were aligned to the reference with Burrows-Wheeler Aligner- Maximum Exact Matches (BWA-MEM) (v0.7.17), and alignment file filtering with Picard (http://broadinstitute.github.io/picard/). The GATK4 pipeline (v4.2.5.0) was used for variant calling and to generate a multi-fasta from a combined variant calling file using Bcftools (v1.14), and finally phylogenetic trees were drawn using fasttree (v2.1.10), quicksnp (v1.0.1) (https://github.com/k-florek/QuickSNP), iqtree (v2.1.4), and rapidnj (v2.3.2).^15–24^

The genome of UOFS Y-4024 was assembled using SPAdes genome assembler (v3.14.1) with default parameters. Quality metrics for the assembly were assessed using QUAST (v5.0.2)^25^, and completeness was evaluated with Benchmarking Universal Single-Copy Orthologs (BUSCO) using the fungal_odb10 dataset.^26^ To annotate the assembly, gene features were transferred from the *C. auris* Clade III reference genome GCF_003013715.1 using Liftoff (v1.6.3).^27^ Transcripts were extracted and translated into proteins using SeqKit (v2.2.0).^19^ In order to determine the mating-type, proteins from LGST01000043.1 were queried against the assembly using tBLASTn (v2.10.1+),^28^ and alignment results were inspected for order and orientation.

### Phylogenetic placement within Clade III

To contextualise UOFS Y-4024 within Clade III of *C. auris*, SRA data corresponding to 566 Clade III genomes—previously curated by Cancino-Muñoz and co-workers^29^ were obtained from NCBI. A core-genome SNP alignment was generated using SNIPPY (v4.6.0), using the Clade III reference strain B11221 (accession CP126632.2) as the reference genome. Repetitive and low-complexity regions were identified using RepeatMasker (v4.1.6) and masked using the remove_blocks_from_aln.py script from the Sanger Institute (https://github.com/sanger-pathogens/remove_blocks_from_aln). Phylogenetic reconstruction was performed with Gubbins using RAxML (model GTRGAMMA, 200 bootstrap replicates).^30^

### Antifungal resistance marker identification

To identify well-known resistance-associated genes/proteins, the proteome of isolate UOFS Y-4024 was queried with protein sequences ERG11 (XP_716761.1) and FKS1 (XP_028893355.2) using BLASTp (v2.10.1+).^28^ Additionally, the predicted proteome was scanned with HMMER3’s hmmscan tool (v3.3.1) against the ResFungi database of fungal resistance profiles compiled by de Carvalho and co-workers.^31^

### Salt tolerance determination

*C. auris* UOFS Y-4024 was inoculated into 5 ml Yeast Potato Dextrose (YPD) media (10 g/l yeast extract, 20 g/l peptone, and 20 g/l d-glucose) and incubated for 24 h at 37°C while shaking. A 10x cell dilution was prepared with YPD media, and the OD_600_ was measured with a Jenway 6400 spectrophotometer. Cells were standardised to an OD_600_ of 0.8. A 10X serial dilution series was prepared for the standardised cells, and 10 µl of the 10^-1^ to 10^-4^ dilutions were spotted onto prewarmed YPD plates supplemented with NaCl concentrations of 0%, 10%, 15%, and 20%. These plates were incubated at 37°C for 72 h. *C. auris* B11221 (Clade III) and *Candida albicans* SC5314 were included as control strains.

### Temperature tolerance determination

*C. auris* UOFS Y-4024 was grown and standardised to an OD_600_ of 0.8, and 200 µl of the standardised cells were plated into a 96-well plate with YPD negative controls. Growth at 30°C, 40°C, and 50°C was monitored for 24 h in a Victor Nivo plate reader. This was done in triplicate and each 96-well plate contained five technical repeats.

### Antifungal susceptibility testing

Antifungal susceptibility, determined by the minimum inhibitory concentration (MIC), was assessed using the AST-YS08 card on the Vitek 2 Compact system (Biomerieux, France), in accordance with the manufacturer's guidelines. The antifungal panel included fluconazole, voriconazole, caspofungin, micafungin, amphotericin B, and flucytosine. Tentative breakpoints as suggested by the Centers for Disease Control and Prevention (CDC) were used in the interpretation of MIC obtained.

### Disinfectant susceptibility testing

*C. auris* UOFS Y-4024 was grown in YPD at 37°C overnight while shaking and standardised to 10^6^ cells/ml. A 2x serial dilution of each disinfectant was prepared in 2x RPMI-1640 of in a 96-well plate. Cells were then exposed to the disinfectant for 20 min and reinoculated in 2x RPMI-1640 to determine MIC values. The disinfectants used were absolute ethanol and commercial preparations containing either 10% povidone iodine, 3.5% sodium hypochlorite, 12% polydimethylammonium chloride, 80% didecyldimethylammonium chloride, 50% benzalkonium chloride, 5% chlorhexidine gluconate, or a combination of 0.5% chlorhexidine gluconate and 70% isopropyl alcohol.

## Results

Three *C. auris* strains were isolated from three different adult locusts, two of which also harboured *Candida orthopsilosis* strains (Table 1). We were able to revive one of the *C. auris* strains (17C2 = UOFS Y-4024) from cryostorage, and genotypic and phenotypic analyses were performed on this strain. This strain was also deposited in the Westerdijk Fungal Biodiversity Institute as CBS 19369.

**Table 1.** Potentially pathogenic yeasts isolated from the alimentary canal of *Locustana paradalina*.

Strain number	Species name	Isolated from
2A2	*Candidozyma* (*Candida*) *auris*	Locust #2 foregut
2B1	*Candida orthopsilosis*	Locust #2 midgut
15C2	*Candidozyma* (*Candida*) *auris*	Locust #15 hindgut
15B1	*Candida orthopsilosis*	Locust #15 midgut
17C2 UOFS Y-4024	*Candidozyma* (*Candida*) *auris*	Locust #17 hindgut

Assembly of *C. auris* UOFS Y-4024 using short-read data and SPAdes default parameters, produced a genome with a total length of 12 369,881 bp across 109 contigs larger than 200 bp, with the largest contig measuring 2  893 009 bp and an N50 of 1  075 510 bp. Assessment of genome completeness using BUSCO indicated 48 missing BUSCOs (Table 2), compared to 58 missing in the Clade III reference genome B11221. These results suggest comparable completeness between the *de novo* assembly and the established reference. The whole genome sequence was deposited in NCBI BioProject database (BioProject ID: PRJNA1214514).

**Table 2.** Genome assembly quality metrics and other characteristics.

Assembly metrics	Value
Contigs > 200bp	109
Total length in bp (contigs > 200 bp)	12 369 881
Largest Contig	2 893 009
N50	1 075 510
L50	4
GC (%)	45.15
*N*'s per 100 000 bp	2.42
Complete BUSCOs	706 (93.1%)
Fragmented BUSCOs	4 (0.5%)
Missing BUSCOs	48 (6.4%)

**Figure 1.**
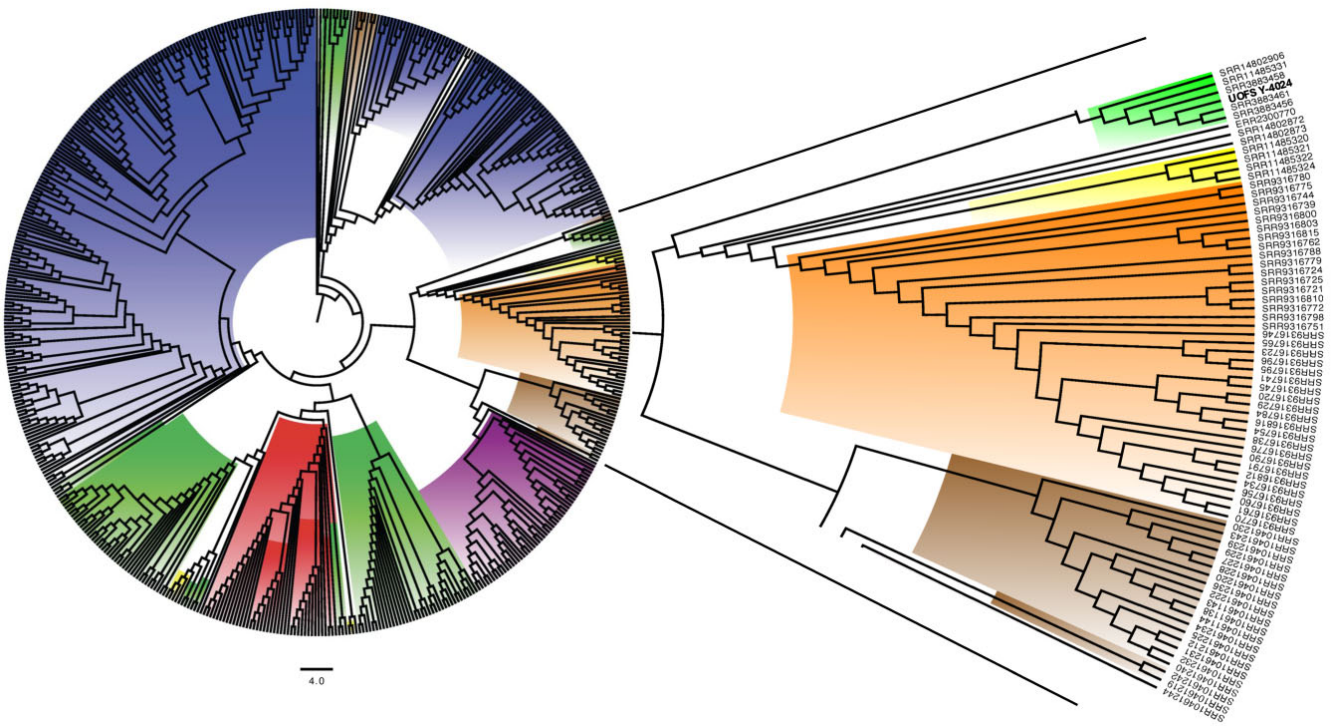
Global phylogeny of all Clade III *Candidozyma auris* isolates analysed (*n* = 566). Coloured nodes indicate the origin country: yellow—Australia; light blue—Austria; black—Canada; orange—China; brown—Kenya; green—South Africa; red—Spain; purple—United Kingdom; and blue—United States.

Phylogenetic placement of UOFS Y-4024 amongst 566 clade III isolates identified the most-closely related strains to have been isolated from South African clinical specimens and sequenced between 2012 and 2014^32^ (Fig. 1). The order and orientation of mating-locus genes were in accordance with a Matα conformation, which is the most common conformation amongst Clade III strains.^33^ The tree file is available at https://figshare.com/articles/dataset/Phylogenetic_Tree_File/29222603?file=55075763.

**Figure 2.**
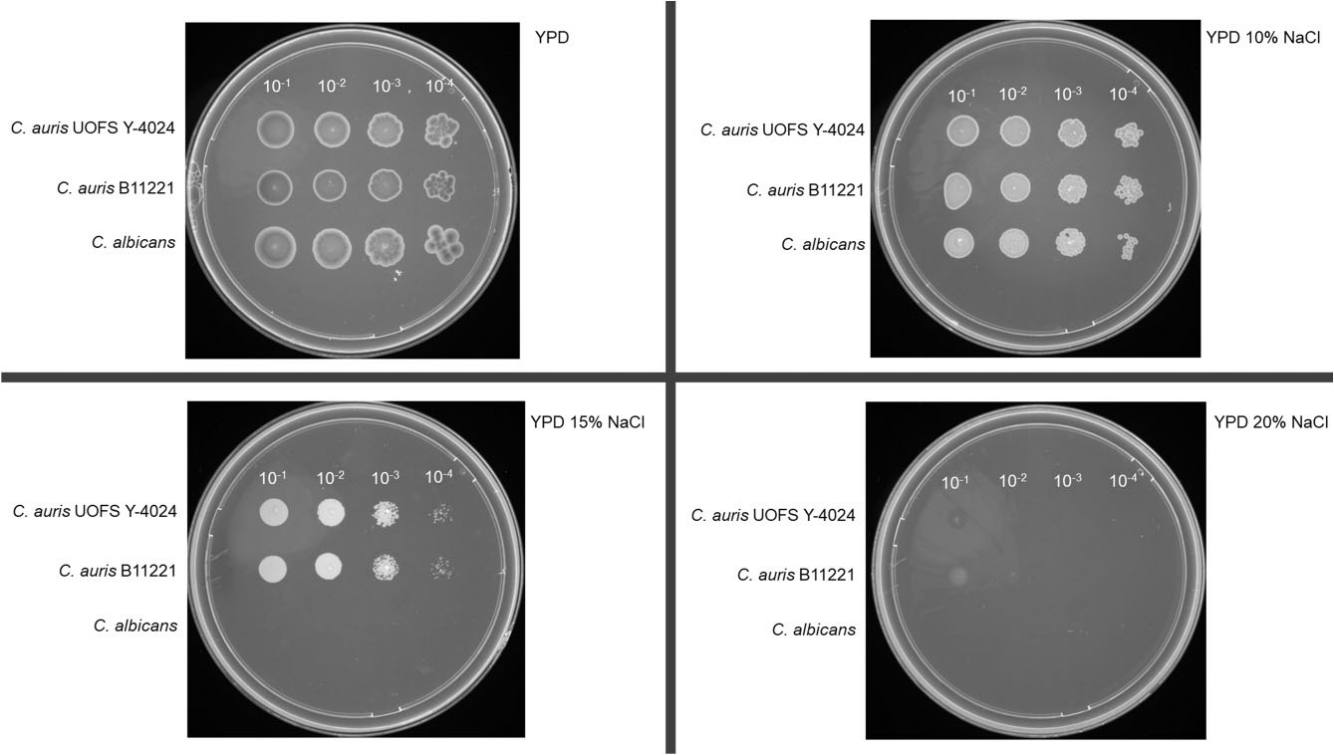
Growth of *C. auris* UOFS Y-4024, *C. auris* B11221 and *C. albicans* SC5314 on media containing NaCl concentrations ranging from 0% to 20%.

From the genome sequence, *FKS1* was identified as wild-type, while *ERG11* carried the V125A/F126L substitution. Phenotypic characterisation and interpretation according to CDC tentative breakpoints confirmed that *C. auris* UOFS Y-4024 is resistant to fluconazole, with an MIC of ≥32 µg/ml. This is also significantly higher than the ≥4 µg/ml point that may indicate fluconazole resistance in *C. auris* as determined by Vitek 2.^34^ Interestingly, although 63 putative resistance markers to 5-fluorocytosine, azoles, caspofungin, fluconazole, itraconazole, micafungin and voriconazole were identified through ResFungi HMM scanning (Supplementary Table S1), this strain was susceptible to the other antifungals tested (Table 3). The isolate was also evaluated against disinfectants from multiple classes and found to be inhibited by all of them at concentrations lower than the recommended use concentration, including a range of quaternary ammonium compounds (QACs) (Table 4). *C. auris* UOFS Y-4024 is halotolerant and can grow on 15% NaCl (Fig. 2) and grows faster at 40°C, with a µ_max_ of 0.061h^-1^ after 120 min, compared to 30°C, where a lower µ_max_ of 0.044h^-1^ was obtained after 210 min. Interestingly, it survived and grew slowly (µ_max_ = 0.009h^-1^) at 50°C. This level of growth was attained after 210 min and maintained for an additional 240 min.

**Table 3.** Minimum inhibitory concentrations (MICs) of antifungal drugs against *C. auris* UOFS Y-4024.

Antifungal drug	Tentative breakpoints (µg/ml)^a^	MIC (µg/ml)
Fluconazole	≥32	32
Voriconazole	No breakpoint^b^	1
Caspofungin	≥2	0.25
Micafungin	≥4	≤0.06
Amphotericin B	>2	≤0.25
Flucytosine	No breakpoint	≤1

a Tentative breakpoints by the CDC (https://www.cdc.gov/candida-auris/hcp/laboratories/antifungal-susceptibility-testing.html).

b Fluconazole is recommended as a surrogate.

**Table 4.** Minimum inhibitory concentrations (MIC_90_) of disinfectants against *C. auris* UOFS Y-4024 after 20 min contact time.

Active compound	MIC_90_ (% final concentration of active compound)	Recommended use concentration of active compound (%)	MIC_90_ ≥ recommended use concentration
Ethanol	29.5	60–80	No
10% povidone iodine	0.096	5–10	No
3.5% sodium hypochlorite	0.24	0.1–0.5	No
12% polydimethylammonium chloride	0.006	0.1–0.5	No
80% didecyldimethylammonium chloride	0.0012	0.05-0.2	No
50% benzalkonium chloride	0.003	0.05–0.2	No
0.05% chlorhexidine gluconate	0.0015	0.5–2	No
0.5% chlorhexidine gluconate combined with 70% ethanol	0.0125 chlorhexidine gluconate0.175 ethanol	0.5–2 chlorhexidine gluconate60%–80% ethanol	No

## Discussion

This study highlights for the first time the presence of *C. auris* in the digestive tract of the brown locusts, *Locustana pardalina*, and shows their potential in disseminating this emerging pathogen. The fact that we were able to isolate *C. auris* from three locusts (15% of locusts tested) using non-selective media and a non-restrictive temperature of 30°C may indicate that *C. auris* is abundant in the locusts and that specific selective isolation is not mandatory.

Interestingly, although *C. orthopsilosis* was only isolated from the midgut, *C. auris* was isolated from the fore- and hindgut. Isolation from the foregut, which is dedicated to food intake and storage, filtering, and partial digestion, indicates that *C. auris* was probably obtained by the locusts via feeding activities. Isolation from the hindgut confirms that *C. auris* can survive the digestive processes in the midgut and is likely to be released back into the environment via frass (faeces).^35^ As indicated, *L. pardalina* is typically exposed to high temperatures and has a preferred body temperature of 39–41°C.^9^ Locusts (*Schistocerca* or *Locusta* spp.) also require between 1 and 2% salts in their diet and can tolerate excess salts (up to 11%) and wide variations in the ratios of ions in the diet.^36^*Locustana pardalina* hoppers are also attracted to NaCl and NaH_2_PO_4_, which may be consumed, possibly to alleviate mineral deficiencies in their grass diet.^37^ Thus, the ability of *C. auris* UOFS Y-4024 to grow under high salt concentration and high temperatures correlates with the environmental and physiological tolerance of locusts. Although the soil and groundwater in the area where the locusts were collected are not particularly high in sodium salts and are considered slightly saline (200–400 mS/m),^38,39^*L. pardalina* can travel up to 2.8 km per day (depending on the instar), while the flying adults can cross large distances.^9^ It is unknown how far they may have migrated during their swarming behaviour or where they may have ingested the yeasts isolated from them. The fact that this strain belongs to Clade III and is closely related to clinical isolates obtained from patients more than a decade ago raises interesting questions regarding the ecology and epidemiology of *C. auris* in South Africa. This and other studies^40^ highlight the importance of insects as potential vectors for the distribution of pathogenic yeasts.

The decreased susceptibility to fluconazole is a common feature of many *C. auris* strains, including ones from non-clinical environments. For example, a *C. auris* strain isolated from an Egyptian cobra from a Moroccan market (also a hot, arid environment) was also considered fluconazole resistant.^41^ Currently, no standards or recommended methods exist to eradicate *C. auris* from contaminated surfaces and infected individuals. Previously, ethanol has been shown to be effective against *C. auris* at a concentration of 70%^42^, and our results support this finding as the MIC_90_ determined was 29.5%. Similarly, povidone iodine at a final concentration of 0.096% effectively inhibited growth of *C. auris* UOFS Y-4024. This is similar to findings by Abdolrasouli and co-workers^43^, who reported MICs between 0.07% and 1.25%. The growth of *C. auris* UOFS Y-4024 was also inhibited by 0.24% sodium hypochlorite. Interestingly, disinfectants containing 0.05% sodium hypochlorite are effective against Clade I *C. auris* but not Clade IV.^44^ We also found that chlorhexidine, either alone or combined with ethanol, is able to inhibit growth of C. auris UOFS Y-4024 at concentrations below the recommended use concentrations.^42,45,46^ Different QACs were also tested and found to be very effective at low concentrations. This observed sensitivity to QACs is interesting, as many studies have indicated that *C. auris* is not susceptible to this class of compound.^44,47,48^ However, it is known that contact time may influence the effect of different disinfectants^47^, making it difficult to compare results between studies. The use of 20 min of contact time in our study may have decreased the MICs compared to those reported in literature. The sensitivity to a range of disinfectants, including ethanol and QACs, enables practical approaches to containment, although the possibility of acquired resistance to these disinfectants is concerning.

**Figure 3.**
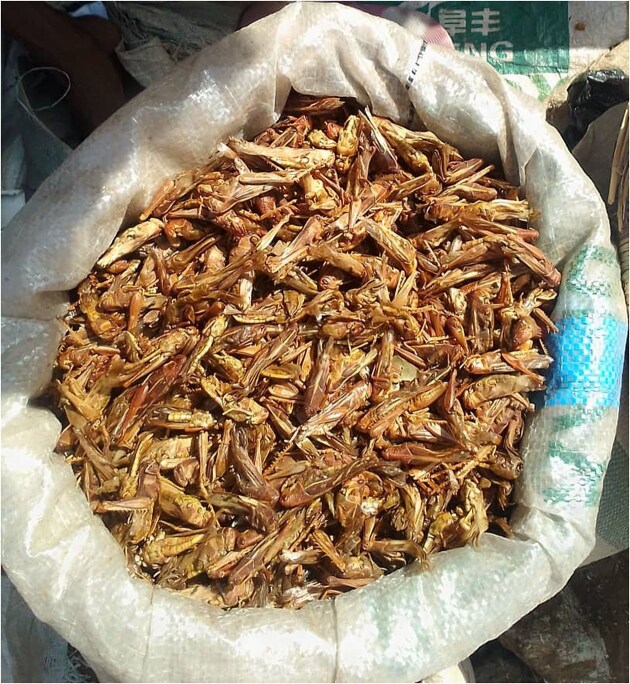
Bag of locusts for sale as food in a market in Nigeria.

In conclusion, the ability of *C. auris* to survive various environmental and chemical stressors in the digestive system of locusts highlights the importance of understanding the interaction between insects and pathogenic yeasts and the possible role of insects in the emergence of new pathogenic yeasts under different ecological regimes. This One Health approach is especially important as locusts (and other insects) are often harvested as food source in many parts of the world (Fig. 3).

## Supplementary Material

myaf069_Supplemental_File Supplementary Table S1. List of genomes with Clade designation and Sequence Read Archive identifications used for initial phylogenetic analyses.

## Data Availability

The whole genome sequence assembly of *Candida auris* UOFS Y-4024, has been deposited in the NCBI BioProject database under the accession number PRJNA1214514. The complete list of publicly available Sequence Read Archive (SRA) data for the 566 Clade III genomes retrieved from NCBI is as published^33^ and available in Supplementary Table S1. The phylogenetic tree file generated from these data is available at Figshare: https://figshare.com/articles/dataset/Phylogenetic_Tree_File/29222603?file=55075763.
